# Supplementary material for: Immunoinformatics Approach to Design Multi-Epitope- Subunit Vaccine against Bovine Ephemeral Fever Disease
Source: Vaccines (Basel). 2021 Aug 19;9(8):925. doi: 10.3390/vaccines9080925 (PMC8402647; doi:10.3390/vaccines9080925)
Supplement: Supplementary file 1 [file vaccines-09-00925-s001.zip › vaccines-1169229-supplementary.pdf]

Supplementary figures

Figure S1. Represents secondary structure of MEV-BEFV peptide as analysed through CFSSP: Chou and Fasman secondary structure prediction server.

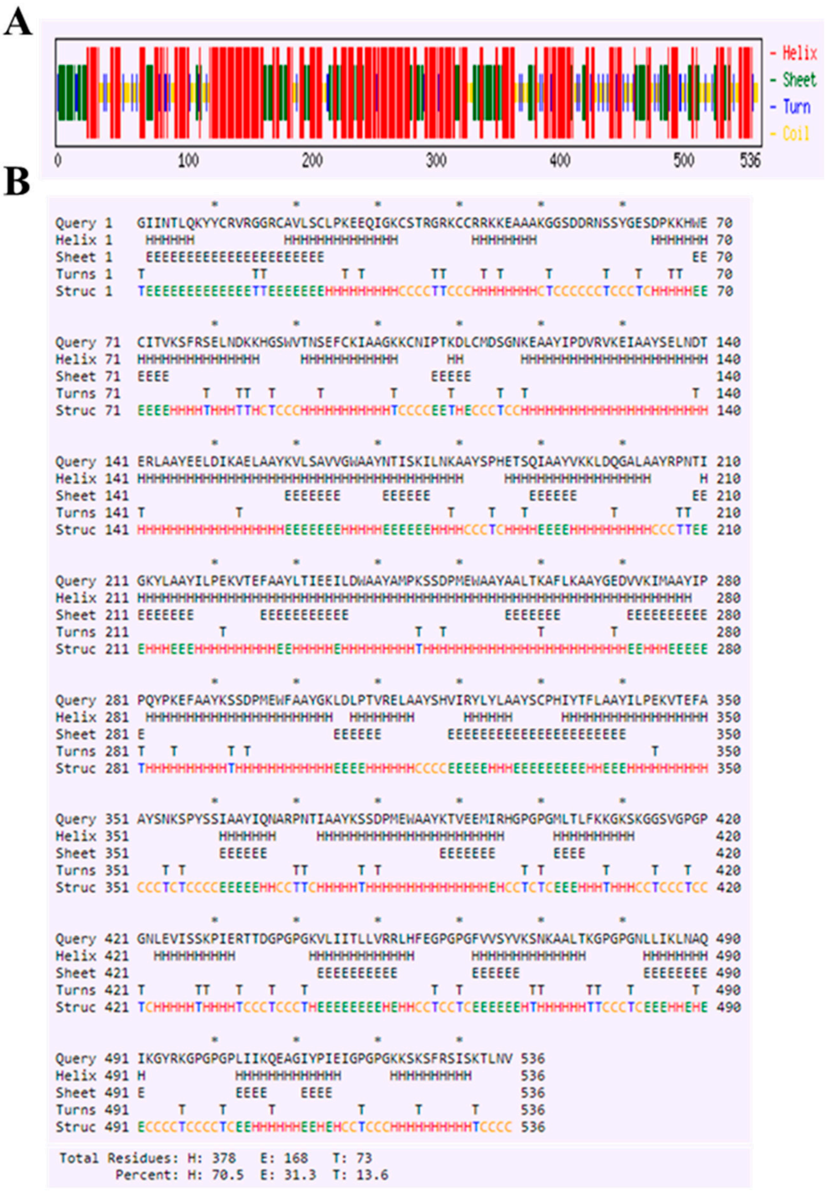

Fig S2. Molecular Dynamics simulation. A. Temperature graph at 300K **B**. Pressure (at) 1 bar pressure

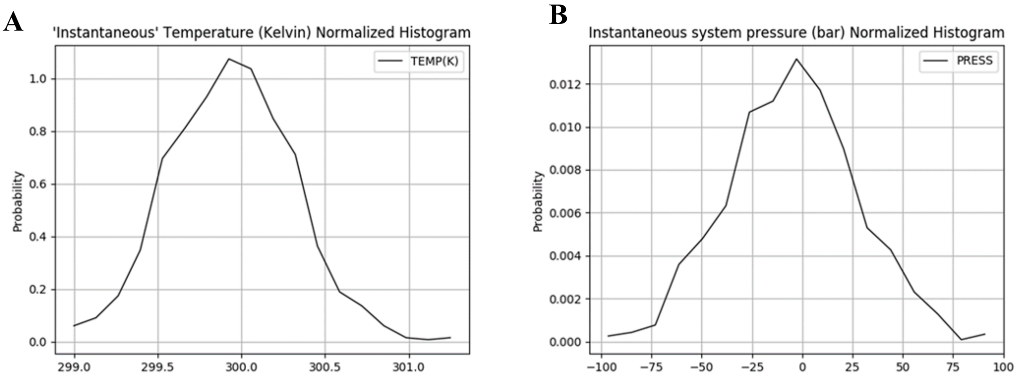

Supplementary Tables

Table S1. Representing the list of all the BoLA- I and BoLA-II alleles (BoLA-DRB3 subtypes) explored during the process for epitope prediction

S.no.	MHC-I alleles	MHC-II alleles
1	BoLA-1:00901	BoLA-DRB3*0101
2	BoLA-1:00902	BoLA-DRB3*0201
3	BoLA-1:01901	BoLA-DRB3*0301
4	BoLA-1:02001	BoLA-DRB3*03021
5	BoLA-1:02101	BoLA-DRB3*0303
6	BoLA-1:02301	BoLA-DRB3*0401
7	BoLA-1:02801	BoLA-DRB3*0501
8	BoLA-1:02901	BoLA-DRB3*0502
9	BoLA-1:03101	BoLA-DRB3*0503
10	BoLA-1:03102	BoLA-DRB3*0601
11	BoLA-1:04201	BoLA-DRB3*0701
12	BoLA-1:04901	BoLA-DRB3*0801
13	BoLA-1:06101	BoLA-DRB3*0901
14	BoLA-1:06701	BoLA-DRB3*0902
15	BoLA-1:07401	BoLA-DRB3*1001
16	BoLA-2:00501	BoLA-DRB3*1002

17	BoLA-2:00601	BoLA-DRB3*1101
18	BoLA-2:00602	BoLA-DRB3*1103
19	BoLA-2:00801	BoLA-DRB3*1201
20	BoLA-2:00802	BoLA-DRB3*1301
21	BoLA-2:01201	BoLA-DRB3*14011
22	BoLA-2:01601	BoLA-DRB3*14012
23	BoLA-2:01602	BoLA-DRB3*1501
24	BoLA-2:01801	BoLA-DRB3*1601
25	BoLA-2:01802	BoLA-DRB3*1602
26	BoLA-2:02201	BoLA-DRB3*1701
27	BoLA-2:02501	BoLA-DRB3*1702
28	BoLA-2:02601	BoLA-DRB3*1703
29	BoLA-2:02602	BoLA-DRB3*1801
30	BoLA-2:02603	BoLA-DRB3*1802
31	BoLA-2:03001	BoLA-DRB3*1901
32	BoLA-2:03202	BoLA-DRB3*1902
33	BoLA-2:04301	BoLA-DRB3*1903
34	BoLA-2:04401	BoLA-DRB3*20011
35	BoLA-2:04402	BoLA-DRB3*20012
36	BoLA-2:04501	BoLA-DRB3*2002
37	BoLA-2:04601	BoLA-DRB3*2003
38	BoLA-2:04701	BoLA-DRB3*2004
39	BoLA-2:04801	BoLA-DRB3*2005
40	BoLA-2:05401	BoLA-DRB3*2101
41	BoLA-2:05501	BoLA-DRB3*2201
42	BoLA-2:05601	BoLA-DRB3*2202
43	BoLA-2:05701	BoLA-DRB3*2301
44	BoLA-2:06001	BoLA-DRB3*2401
45	BoLA-2:06201	BoLA-DRB3*2402
46	BoLA-2:06901	BoLA-DRB3*25011
47	BoLA-2:07001	BoLA-DRB3*25012
48	BoLA-2:07101	BoLA-DRB3*2601
49	BoLA-3:00101	BoLA-DRB3*2702
50	BoLA-3:00102	BoLA-DRB3*2703
51	BoLA-3:00103	BoLA-DRB3*2704

52	BoLA-3:00201	BoLA-DRB3*2705
53	BoLA-3:00401	BoLA-DRB3*2707
54	BoLA-3:00402	BoLA-DRB3*2708
55	BoLA-3:00403	BoLA-DRB3*2709
56	BoLA-3:01001	BoLA-DRB3*2710
57	BoLA-3:01101	BoLA-DRB3*2801
58	BoLA-3:01701	BoLA-DRB3*2901
59	BoLA-3:01702	BoLA-DRB3*3001
60	BoLA-3:01703	BoLA-DRB3*3002
61	BoLA-3:02701	BoLA-DRB3*3101
62	BoLA-3:02702	BoLA-DRB3*3103
63	BoLA-3:03501	BoLA-DRB3*3201
64	BoLA-3:03601	BoLA-DRB3*3202
65	BoLA-3:03701	BoLA-DRB3*3203
66	BoLA-3:03801	BoLA-DRB3*3301
67	BoLA-3:05001	BoLA-DRB3*3401
68	BoLA-3:05002	BoLA-DRB3*3402
69	BoLA-3:05101	BoLA-DRB3*3501
70	BoLA-3:05201	BoLA-DRB3*3601
71	BoLA-3:05301	BoLA-DRB3*4001
72	BoLA-3:05801	BoLA-DRB3*4101
73	BoLA-3:05901	BoLA-DRB3*4201
74	BoLA-3:06501	BoLA-DRB3*4301
75	BoLA-3:06601	BoLA-DRB3*4302
76	BoLA-3:06602	BoLA-DRB3*4303
77	BoLA-3:06801	BoLA-DRB3*4401
78	BoLA-3:07301	BoLA-DRB3*4501
79	BoLA-4:02401	BoLA-DRB3*46011
80	BoLA-4:02402	BoLA-DRB3*4701
81	BoLA-4:06301	BoLA-DRB3*4802
82	BoLA-5:00301	BoLA-DRB3*5701
83	BoLA-5:03901	BoLA-DRB3*5702
84	BoLA-5:06401	BoLA-DRB3*5801
85	BoLA-5:07201	BoLA-DRB3*5901
86	BoLA-6:01301	BoLA-DRB3*6001

87	BoLA-6:01302	BoLA-DRB3*6101
88	BoLA-6:01401	BoLA-DRB3*6201
89	BoLA-6:01402	BoLA-DRB3*6301
90	BoLA-6:01501	BoLA-DRB3*6401
91	BoLA-6:01502	BoLA-DRB3*6402
92	BoLA-6:03401	BoLA-DRB3*6501
93	BoLA-6:04001	BoLA-DRB3*6601
94	BoLA-6:04101	BoLA-DRB3*0101
95	BoLA-AW10	BoLA-DRB3*0201
96	BoLA-D18.4	BoLA-DRB3*0301
97	BoLA-HD6	BoLA-DRB3*03021
98	BoLA-JSP.1	BoLA-DRB3*0303
99	BoLA-T2a	BoLA-DRB3*0401
100	BoLA-T2b	BoLA-DRB3*0501
101	BoLA-T2c	BoLA-DRB3*0502
102	BoLA-T5	BoLA-DRB3*0503
103	BoLA-T7	BoLA-DRB3*0601
104	BoLA-amani.1	
105	BoLA-gb1.7	

Table S2. Epitope conservancy for all the prioritised predicted epitopes of the MEV-BEFV

Epitope sequence	Epitope length	Epitope type	Percent of protein sequence matches at identity >= 80%	Minimum identity	Maximum identity
**Matrix protein**					
MLTLFKKGKSKGGSV	15	HTL	**50.00% (4/8)**	93.33%	100.00%
NLEVISSKPIERTTD	15	HTL	**87.50% (7/8)**	93.33%	100.00%
GGSVDDRNSSYGESDP	16	BCL	**100.00% (8/8)**	93.75%	100.00%
**Glycoprotein**					
HWECITVKSFRSELND	16	BCL	**50.00% (4/8)**	93.75%	100.00%
IPDVRVKEI	9	CTL	**87.50% (7/8)**	88.89%	100.00%
EELDIKAEL	9	CTL	**100.00% (8/8)**	100.00%	100.00%
KVLSAVVGW	9	CTL	**100.00% (8/8)**	100.00%	100.00%
NTISKILNK	9	CTL	**100.00% (8/8)**	100.00%	100.00%
SPHETSQI	8	CTL	**87.50% (7/8)**	75.00%	100.00%
VKKLDQGAL	9	CTL	**100.00% (8/8)**	100.00%	100.00%
KVLIITLLVRRLHFE	15	HTL	**37.50% (3/8)**	66.67%	100.00%
**Nucleoprotein**					
RPNTIGKYL	9	CTL	**100.00% (8/8)**	100.00%	100.00%
ILPEKVTEF	9	CTL	**100.00% (8/8)**	100.00%	100.00%
LTIEEILDW	9	CTL	**100.00% (8/8)**	88.89%	100.00%
AMPKSSDPMEW	11	CTL	**100.00% (8/8)**	100.00%	100.00%
AALTKAFLK	9	CTL	**100.00% (8/8)**	100.00%	100.00%
GEDVVKIM	8	CTL	**100.00% (8/8)**	100.00%	100.00%
IPPQYPKEF	9	CTL	**100.00% (8/8)**	100.00%	100.00%
KSSDPMEWF	9	CTL	**100.00% (8/8)**	100.00%	100.00%
GKLDLPTVREL	11	CTL	**100.00% (8/8)**	90.91%	100.00%
SHVIRYLYL	9	CTL	**100.00% (8/8)**	100.00%	100.00%
SCPHIYTFL	9	CTL	**100.00% (8/8)**	100.00%	100.00%
ILPEKVTEF	9	CTL	**100.00% (8/8)**	100.00%	100.00%
SNKSPYSSI	9	CTL	**100.00% (8/8)**	100.00%	100.00%
IQNARPNTI	9	CTL	**100.00% (8/8)**	100.00%	100.00%
IQNARPNTI	9	CTL	**100.00% (8/8)**	100.00%	100.00%
HGSWVTNSEFCKIAAG	16	BCL	**100.00% (8/8)**	100.00%	100.00%
FVVSYVKSNKAALTK	15	HTL	**100.00% (8/8)**	100.00%	100.00%
NLLIKLNAQIKGYRK	15	HTL	**100.00% (8/8)**	100.00%	100.00%
**Phosphoprotein**					
KTVEEMIRH	9	CTL	**87.50% (7/8)**	77.78%	100.00%
CNIPTKDLCMDSGNKE	16	BCL	**37.50% (3/8)**	50.00%	100.00%
PLIIKQEAGIYPIEI	15	HTL	**100.00% (8/8)**	80.00%	100.00%
KKSKSFRSISKTLNV	15	HTL	**87.50% (7/8)**	73.33%	100.00%
